# An itchy erythematous papular skin rash as a possible early sign of COVID-19: a case report

**DOI:** 10.1186/s13256-020-02538-y

**Published:** 2020-11-09

**Authors:** Alice Serafini, Peter Konstantin Kurotschka, Mariabeatrice Bertolani, Silvia Riccomi

**Affiliations:** 1 Azienda USL di Modena, Via Bernardino Ramazzini 90, 41121 Modena MO, Emilia Romagna Region Italy; 2 COVID-19 Special Home Care Unit, Azienda USL di Modena, Via Bernardino Ramazzini 90, 41121 Modena MO, Emilia Romagna Region Italy; 3 Department of Medical Science and Public Health, University of Cagliari, Cagliari, Italy; 4 Regional Health Trust of Sardinia Region, Cagliari, Italy; 5 Section of Dermatology, Department of Clinical and Experimental Medicine, University of Parma, Parma, Italy

**Keywords:** COVID-19, Skin manifestations, Signs and symptoms, Primary healthcare, General practitioners

## Abstract

**Background:**

Several recent studies suggest the possibility of a skin rash being a clinical presentation of coronavirus disease 2019 (COVID-19). The purpose of this case report is to bring attention to skin manifestations in the early stage of COVID-19 in order to support frontline physicians in their crucial activity of case identification.

**Case presentation:**

The patient is an Italian 32-year-old female nurse who had several close contacts with multiple patients with COVID-19 as part of her professional workload. On March 13, 2020, the patient developed an itchy, erythematous papular rash (sparing only her face, scalp, and abdomen), which lasted for 10 days. The rash was accompanied by a feeling of general fatigue that gradually worsened over the following days and has continued for 5 months (until the end of July 2020). During the first week of remote assessment carried out by her general practitioner, the patient gradually developed a dry cough, intermittent fever, and diarrhoea and then had a positive test result for severe acute respiratory syndrome coronavirus 2 (SARS-CoV-2). Her skin manifestations disappeared completely 48 days after the onset of the disease, followed by the disappearance of the dry cough.

**Conclusions:**

In light of recent studies, this case report suggests that skin manifestations, when taken into account with other situational factors (such as profession and patient history) should be taken into proper consideration by frontline physicians as possibly being caused by SARS-CoV-2. Early identification of COVID-19 is a key part of the strategy of case detection and case isolation. To enhance this activity, further research is needed to establish frequency, symptoms, signs, and pathogenesis of skin manifestations in patients with COVID-19.

## Take-home messages

- During the severe acute respiratory syndrome coronavirus 2 (SARS-CoV-2) outbreak, the sudden appearance of a skin rash in a patient with no other etiology to explain its clinical presentation should encourage physicians to consider a possible coronavirus disease 2019 (COVID-19) diagnosis. This is particularly true for high-risk populations, even if, to the best of current knowledge, no specific cutaneous manifestation should be considered pathognomonic for COVID-19.
- The probability of a skin rash being an early sign of an underlying SARS-CoV-2 infection should be evaluated in light of the patient’s epidemiological risk profile and the local epidemiological situation. To this end, it is important to note that skin manifestations are currently reported as rare signs of the disease.
- Further research is needed to improve current knowledge about epidemiology, clinical presentation, and pathogenesis of COVID-19 skin manifestations. This is necessary in order to understand if the sudden appearance of an isolated skin rash could justify the routine prescription of home isolation and/or further patient testing to determine the presence or not of a SARS-CoV-2 infection, especially in primary care settings.

## Background

Coronavirus disease 2019 (COVID-19) is a respiratory illness caused by severe acute respiratory syndrome coronavirus 2 (SARS-CoV-2). It was first identified in Wuhan, a city in the province of Hubei, China, in late December 2019 [1]. On March 11, 2020, the World Health Organization (WHO) declared the SARS-CoV-2 outbreak a pandemic. Since then, it has become a public health emergency of unprecedented proportions, caused major restrictions on the everyday lives of billions of people, and strained healthcare systems worldwide [2]. Italy was the first European country to experience a dramatic increase in the number of cases, starting in February 2020, facing one of the largest clusters of COVID-19 in Europe [3].

Excluding asymptomatic cases, clinical presentations of COVID-19 can range from a mild respiratory infection (common cold–like illness, 80%) to severe pneumonia (14%). In a percentage of these cases, the patient’s pneumonia can degenerate to potentially lethal acute respiratory distress syndrome (5%) [4, 5]. The most commonly reported clinical manifestations of SARS-CoV-2 infection include fever (83–99%), cough (59–82%), fatigue (44–70%), anorexia (40–84%), shortness of breath (31–40%), and myalgias (11–35%). Other, less specific symptoms include sore throat, nasal congestion, headaches, conjunctivitis, and gastrointestinal manifestations, such as nausea and diarrhoea [5–8]. Additionally, according to a recent metanalysis, olfactory and taste disorders have been reported in 44–53% of patients with COVID-19 [9]. These symptoms have assumed increasing importance in the identification of COVID-19 possible cases: the presence of an isolated anosmia, in the United Kingdom, is sufficient to identify the patient as a suspect case and prescribe home isolation and an oropharyngeal swab for the detection of SARS-CoV-2 [10].

Even as a growing number of studies are published detailing the clinical signs of COVID-19, in the words of Giacomelli *et al.*, the spectrum of symptoms catalogued to date may still have failed to “give an account of minor symptoms that may be present at earlier stage of the infection.” [11]*.* As the majority of studies were conducted in a hospital setting, this could result in less attention currently being given to minor symptoms. It should be noted that as of today, no published studies have fully described the disease’s course in a primary care setting. When the first draft of this article was submitted in mid-April 2020, only two prepublished papers suggested that cutaneous manifestations could be present at the onset of COVID-19. The first of these studies was a case report about a patient in Thailand with a petechial skin rash who was later identified as being SARS-CoV-2 positive [12]. The second paper analyzed cutaneous involvement in 88 hospitalized patients with COVID-19 positivity in Lecco, Italy. In this study, the authors found that 20.4% (n = 18) of patients developed cutaneous manifestations and that 44% of them (n = 8) had skin manifestations at the onset of the virus. Authors reported the following types of skin manifestations: erythematous rash and widespread urticarial and chickenpox-like vesicles [13].

Since then, considerable research has been focused on the virus’ rash-related symptoms, and a variety of skin lesions associated with COVID-19 have been described [14–19]. Recently, findings of an international registry from 31 countries, collecting 716 cases of new-onset cutaneous manifestations in patients with confirmed or suspected COVID-19, were published. Authors described the following skin lesions in patients with confirmed COVID-19: morbilliform (22%), pernio-like (18%), urticarial (16%), macular erythema (13%), vesicular (11%), papular-squamous (9.9%), and retiform purpura (6.4%) [19].

In line with recent published studies and the growing evidence about this still largely unexplored topic, the aim of this case study is to report and bring attention to skin manifestations in the early stages of COVID-19 in order to support primary care physicians in their crucial activity of case finding and case isolation [19].

## Case presentation

The patient is a 32-year-old female nurse of Caucasian ethnicity. She works in a private clinic in Emilia Romagna, a region in northern Italy that borders Lombardy. At the time of publication, Emilia Romagna was the Italian region with the second highest total number of confirmed cases and deaths in all of Italy. On March 13, 2020, the first day of her symptoms’ onset, Emilia Romagna had 299 confirmed cases, and by the time of the first submission of this article (April 11, 2020), Italian government data reported a total of 3263 confirmed cases in the region; the latest update of the total number of cases, dated September 8, 2020, reported 32.760 cases [20–22].

**Fig. 1:**
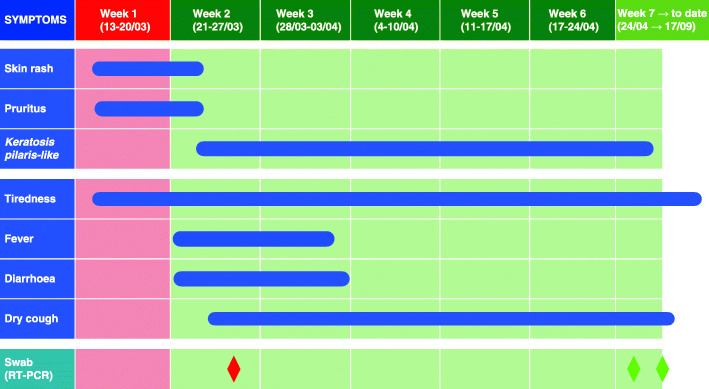
Sequence of the symptoms’ appearance and persistence, their relationship with the patient’s health behaviors, and the timing and results of the oropharyngeal swabs. *Blue lines* show the period of the symptoms’ appearance and persistence. The *red column* covers the period in which the patient carried out normal activities (skin rash and tiredness were the only manifestations of the disease). *Green columns* cover the symptomatic period in which the patient observed home isolation (typical COVID-19 symptoms appeared). *Red diamond* = positive oropharyngeal swab; *green diamonds* = negative oropharyngeal swabs

On March 13, 2020, the patient started to feel mildly tired and presented with an extremely itchy erythematous rash. Small, folliculocentric papules, associated with pruritus, appeared first on the patient’s extremities (hands, feet, forearm, legs, and back surface of the ears) and then spread to her whole body, sparing only her face, scalp, and abdomen (Fig. 1). The itch worsened during the night, making it difficult for her to rest. Psychological factors are known to worsen pruritus [23]. At first, the patient herself associated the development of symptoms with psychological stress and the changes in her working environment. As a result, she continued working without asking for medical advice.

Seven days after the onset of the skin rash, the patient developed a fever (37.5 °C, axillary) and watery diarrhoea (three to four episodes per day). As a result of these developments, she called her general practitioner (GP), who performed a comprehensive remote telephone assessment and identified her as a suspected COVID-19 case. This diagnosis was based on the presence of fever, a typical and well-known symptom of COVID-19, and due to her epidemiological high-risk profile (being a healthcare professional exposed to several patients with known COVID-19).

It should be noted that the patient did not have any chronic disease and had no personal or family history of autoimmune illness, atopy, or other skin problems. She did not smoke or consume alcohol. Furthermore, she did not have any ongoing chronic treatments, nor had she taken any new drugs in the weeks before the symptoms’ onset.

**Fig. 2:**
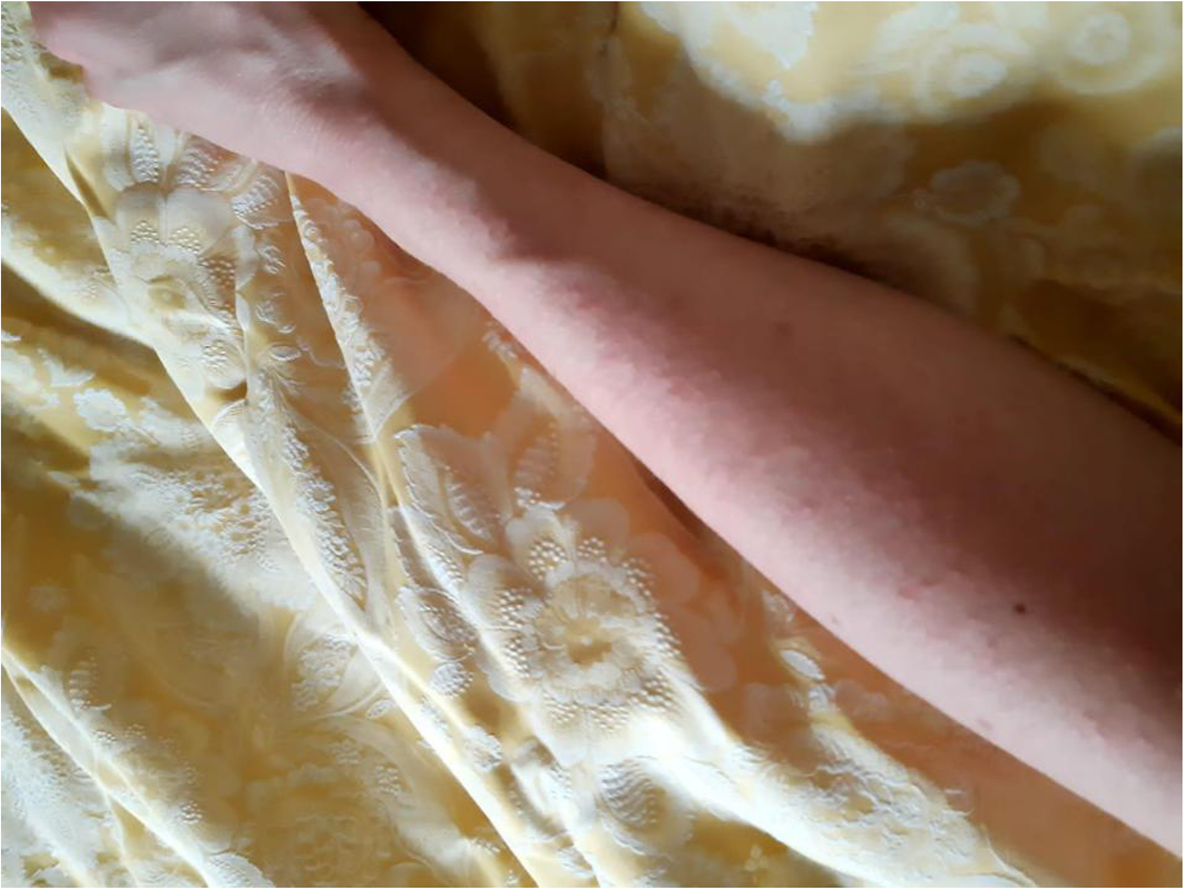
Photo taken by the patient on March 20, 2020, showing papular erythematous rash on the forearm

**Fig. 3:**
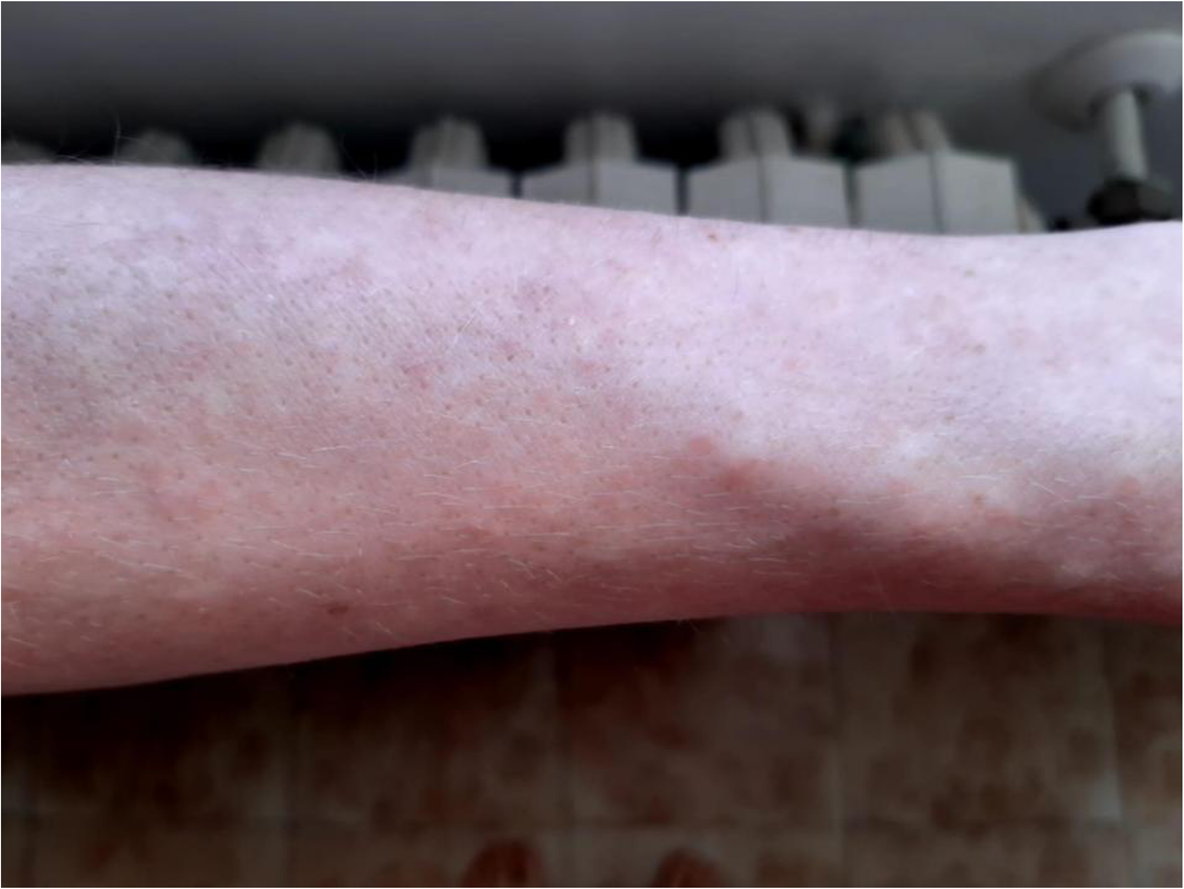
Photo taken by the patient on March 20, 2020, showing papular erythematous rash on the forearms (detail)

The GP evaluated the patient’s skin rash during this first remote consultation via pictures the patient took herself (Figs. 2 and 3). The patient was told to quarantine at home, ensure an appropriate fluid intake, and self-medicate with paracetamol 500 mg if needed to manage the symptoms. To treat the skin rash, her GP prescribed an oral H1-antihistamine (cetirizine 10 mg once per day). As the patient was living alone (she was already living in self-isolation in order to reduce the possibility of infecting her family members), her GP started telemonitoring her case via scheduled follow-up calls at 3-day intervals [24].

During the first week of her remote monitoring, the patient began gradually experiencing a dry cough. The fever showed an intermittent trend during the day, higher in the evening but never higher than 37.5 °C. Her diarrhoea gradually improved (from watery diarrhoea to occasional episodes of unformed stool rushes). The itchy rash improved with the administration of oral H1-antihistamine, disappearing after 10 days.

Thirteen days after the onset of symptoms and 7 days after the appearance of fever, the patient was tested for SARS-CoV-2 infection with an oropharyngeal swab (real-time polymerase chain reaction [RT-PCR]), and her results were positive. During the whole period of remote monitoring, the vital signs of the patient remained stable, and she never reported dyspnoea.

When this article was first submitted, 31 days after the first appearance of the patient’s rash, she was still experiencing a dry cough, headaches, and moderate fatigue. Furthermore, even though she was not experiencing pruritus anymore, she reported the persistence of small papules on her arms, imparting a stippled appearance to the skin resembling gooseflesh, similar to keratosis pilaris. The patient described these lesions as distinctly palpable; however, they were not visible in the remote assessment. These small, palpable papules gradually disappeared over time. During her remote consultation on April 30, 2020, her GP ascertained, for the first time since the onset of the disease, an absence of cutaneous symptoms. The patient then had negative test results of two consecutive oropharyngeal swabs (April 26 and 30, 2020). The dry cough persisted for several more weeks and disappeared only very gradually, finally subsiding completely at the end of May (May 20–22, 2020). At this time, the patient returned to work, although a moderate sense of exhaustion persisted until July 2020. She described herself as completely recovered only at the end of August 2020.

## Discussion

Here, we present a case of an itchy, erythematous papular rash as the first clinical manifestation of COVID-19 and describe the disease course in a young Italian nurse. To the best of our knowledge, this is the only published case of skin manifestations of COVID-19 authored by GPs and entirely managed in primary care.

At the onset of her illness, the patient reported a severe itchy skin rash with pruritus that worsened during the night, making it difficult for her to rest. Scabies, a skin infestation caused by the mite *Sarcoptes scabiei*, is a frequent cause of severe pruritus, where pruritus is due to a delayed hypersensitivity response to the mite proteins [25]. The patient had no history indicating an increased likelihood to contract scabies or the disease-specific linear skin burrows. As a result, scabies was quickly excluded as a diagnosis. Another frequent cause of pruritus is an adverse drug reaction. Almost any drug may induce pruritus by various pathogenic mechanisms [26]. Drug-induced skin manifestations represent a challenging differential diagnosis in patients with COVID-19 with skin manifestations. In fact, different medications have been used to treat COVID-19 in both hospital and outpatient settings, and many of them are known to cause cutaneous side effects. For this reason, it can be hard to establish causation between COVID-19 infection and skin eruptions when dealing with patients who have received these medications [17]. In this case, we can exclude an adverse drug reaction due to the fact that the patient did not receive a COVID-19–specific drug treatment. Moreover, the skin rash was the first manifestation of the disease while the patient had no history of recent or chronic drug intake.

Other common causes of itchy skin rashes could also be reasonably excluded: the patient did not have any chronic physical or mental illnesses, lived in good hygienic conditions, did not use aggressive soaps or cosmetics, was not pregnant, and had no personal or family history of autoimmune illness, atopy, or other skin problems [27].

The occurrence of erythematous and itchy skin lesions linked to COVID-19, like those in our patient’s case, has been reported by several studies [14–19]. Sachdeva *et al.* highlighted that cutaneous manifestations of COVID-19 can range from maculopapular exanthem (as in our patient’s case) to papulovesicular rash, urticaria, painful acral red-purple papules, livedo reticularis lesions, and petechiae. In some cases, these lesions occurred prior to the onset of respiratory symptoms [14].

Also, in a large study from Spain, the most common cutaneous manifestations occurring in 375 patients with confirmed or suspected COVID-19 were maculopapular eruptions (47%) [16]. Unlike the cases reported by the authors, our patient’s rash appeared before the onset of other symptoms and was not associated with a severe episode of the disease. This difference is likely due to the underrepresentation of mild cases of COVID-19 in the study’s Spanish sample.

The results of a large international registry study across 31 countries reported morbilliform rash as the most common morphology of the dermatologic conditions experienced by patients with COVID-19. Consistent with our observations, the majority of reported morbilliform rash cases were pruritic (61%), involved arms and legs (55% and 58%, respectively), and spared the face (79%). In addition, in the majority of cases, the rash was not associated with previous comorbidity nor complications in the disease course [19]. Lastly, a review authored by Wollina *et al.* included maculopapular rash into the proposed categorization of the cutaneous signs of COVID-19 [15].

The occurrence of erythematous and itchy skin lesions linked to COVID-19 have been reported by several studies, supporting our hypothesis of an association between a COVID-19 infection and our patient’s skin rash. The period of communicability of an individual infected with SARS-CoV-2 is still uncertain, as is the relationship between viral load, disease severity, and transmission rate. However, some studies suggest that the viral load is highest shortly after the onset of symptoms [28]. This means that the transmission rate of the infection could be higher in the early stages of the disease, making the early identification of possible cases even more important. Early identification of COVID-19 symptoms and possible cases is part of the evolving strategy of case detection and isolation that, in the current phases of coexistence with SARS-CoV-2, is a crucial part of the activity of primary care providers.

### Strengths and limitations

The present case study suggests that a skin rash could be an early clinical manifestation of COVID-19. The plausibility of the association between our patient’s skin rash and the SARS-CoV-2 infection is supported by several elements: (1) recently published reports of skin manifestations related to COVID-19 are consistent with our observations; (2) the patient had a positive test result of a SARS-CoV-2 oropharyngeal swab by RT-PCR; (3) the patient is a healthcare professional and therefore has a high-risk epidemiological profile; and (4) we could reasonably exclude other causes of her skin rash.

Nevertheless, some limitations should be mentioned. To begin with, the patient is a healthcare professional and thus part of a professional group under enormous social and mental strain due to the pandemic. Being part of a professional group that in a health emergency has the duty to care for ill people and is therefore responsible for public well-being is likely to generate stress and stress-related clinical manifestations [29, 30]. In light of the above-mentioned points, we cannot exclude the possibility that the patient’s psychological stress could have had an influence on the subjective perception of pruritus, worsening it. However, we believe that in the case of our patient, the role of stress in exacerbating her symptoms was limited, and an alternative diagnosis, such as psychogenic itch, is extremely unlikely [31]. The patient did not have any personal or family history of mental illness, and the screening questionnaire performed during the follow up period for major depressive disorder showed only borderline results [32].

Second, the absence of a comprehensive physical examination, due to the shortage of personal protective equipment and the massive move to telemedicine for primary care services, could have limited the diagnostic accuracy of the skin rash. However, teledermatology has been shown to be as effective as in-person care, even if the studies thus far have been focused on those done in a dermatology specialist setting. Further studies are needed to compare remote dermatologic assessment with in-person care in a primary care setting performed by GPs [33, 34].

Third, no further diagnostic steps (for example, referral to a specialist, skin biopsy) were performed. This was due to the fact that during the quarantine/lockdown phase of the pandemic in Italy, both physical contact and the general movement of people were heavily restricted. Every further investigation or test had to be weighed against the risk of spreading the disease, and nonessential tests were not performed during this period. The patient was managed in a general practice setting, where, even in normal times, referral to specialist services, laboratory testing, and skin biopsy are rarely indicated for the management of an uncomplicated skin rash. That said, the authors are aware that this leaves the door open to some diagnostic uncertainty.

Finally, since the patient was in close contact with multiple patients with COVID-19, it has not been possible to establish her exact incubation period. She developed the skin rash a few days after many patients she took care of during her shifts began to experience COVID-19 symptoms and then had a positive test result for SARS-CoV-2. The onset of her symptoms is therefore consistent with the mean incubation period of 5.2 days (95% confidence interval, 4.1 to 7.0) described by Li *et al.* [35].

## Conclusion

The present study is, to the best of our knowledge, the first primary care case report of a female patient who developed a skin rash as the first clinical manifestation of COVID-19. No cutaneous sign can be considered pathognomonic for SARS-CoV-2 infection, and further studies involving larger patient samples are needed to better understand several aspects of the cutaneous involvement of COVID-19. Areas that merit further study include the causal relationship between the infection and skin lesions, the role of pathogenic mechanisms, the absolute frequency and the frequency of skin lesions at the onset, as well as the disease course, the severity, and the transmission rate when skin involvement is the unique manifestation of the COVID-19. More in general, our knowledge regarding the diagnostic accuracy of signs and symptoms to determine if a patient is affected by SARS-CoV-2 infection is still limited at the point that a recent Cochrane review concluded that, “based on currently available data, neither absence nor presence of signs or symptoms are accurate enough to rule in or rule out disease” [36].

This said, the sudden appearance of a skin rash for which other causes could be reasonably excluded should encourage primary care and frontline physicians to consider SARS-COV-2 as a possible underlying diagnosis.

## Data Availability

The data that support the findings of this study are available on request from the corresponding author.
